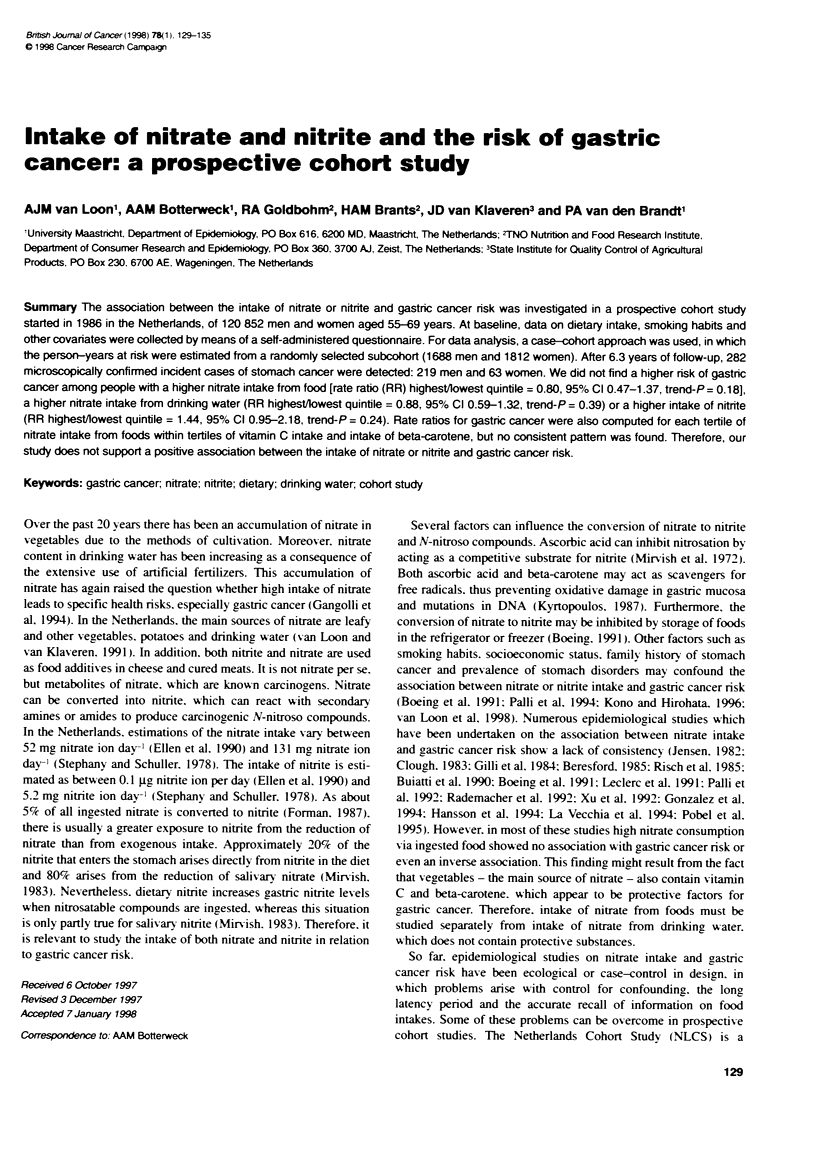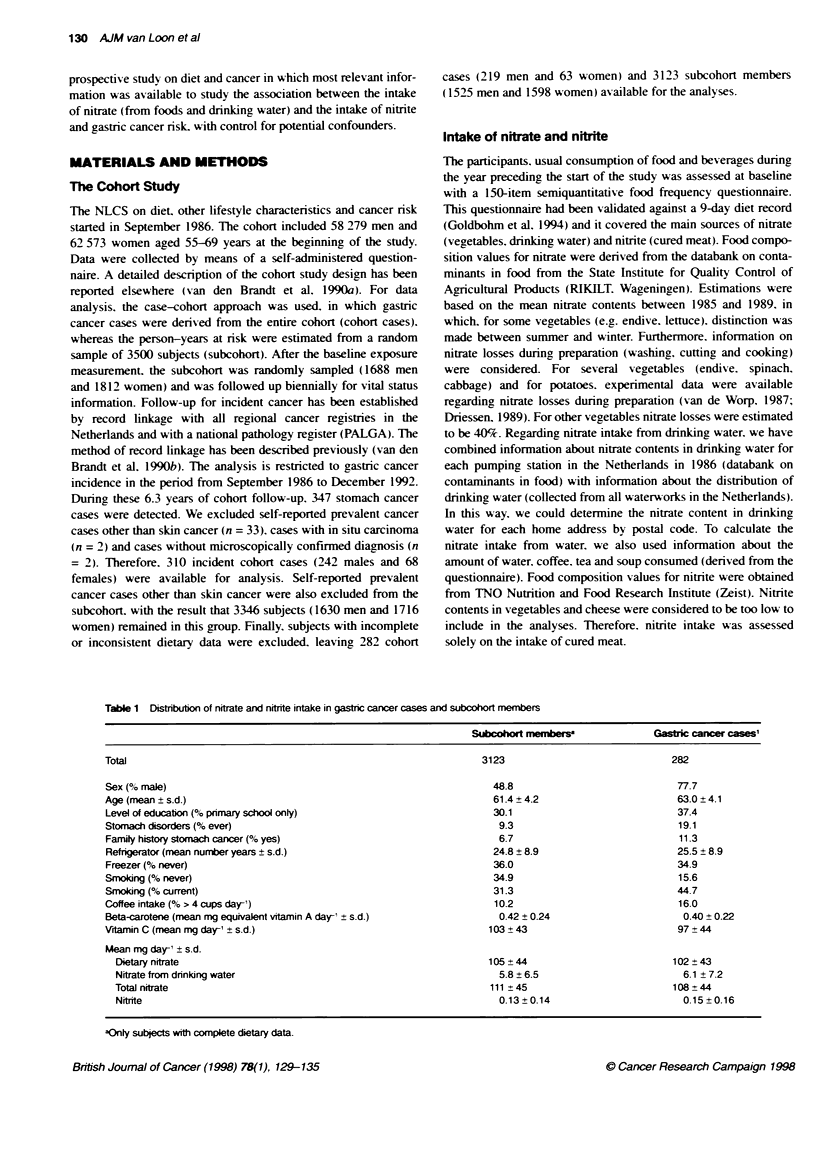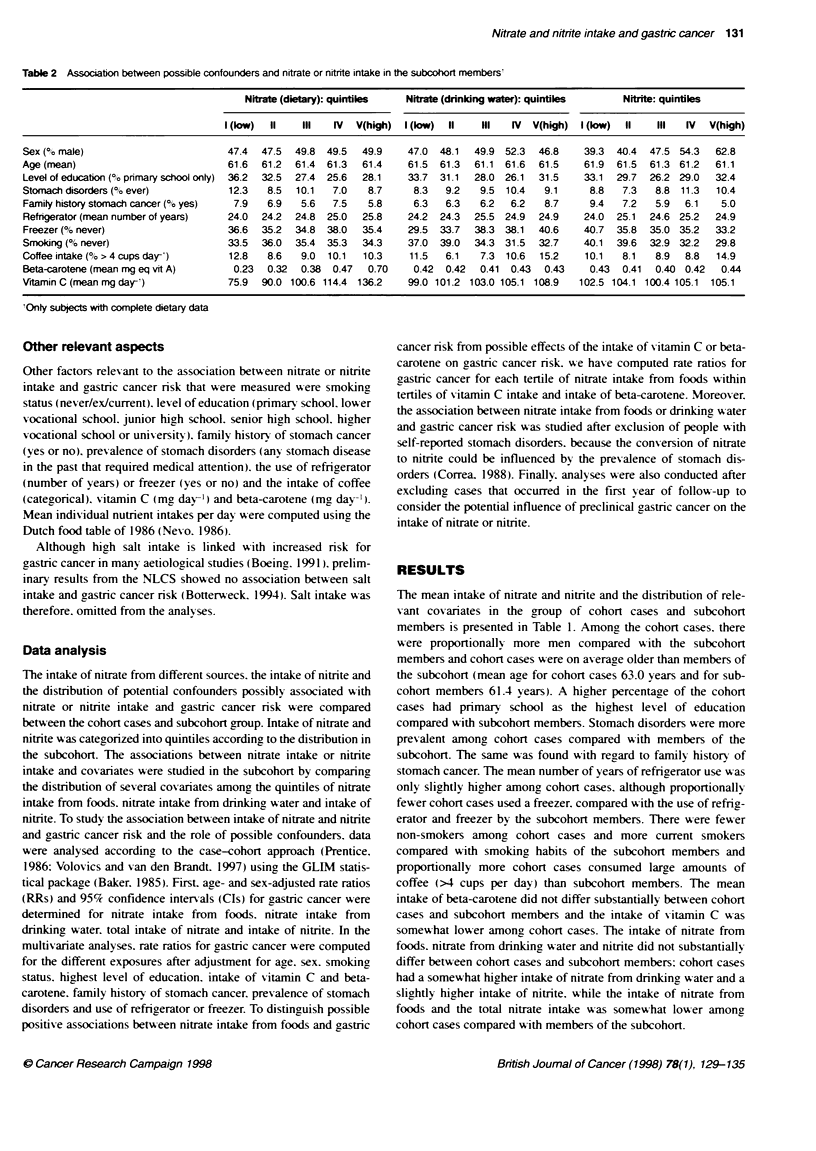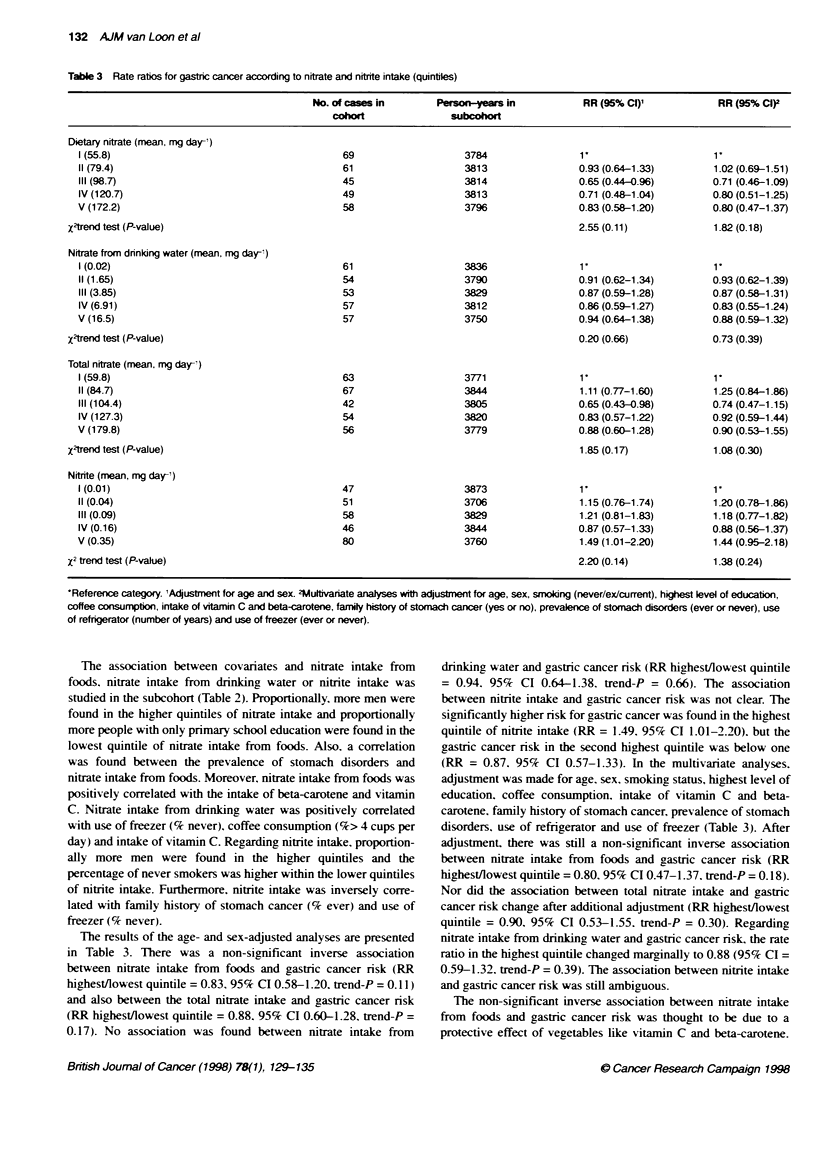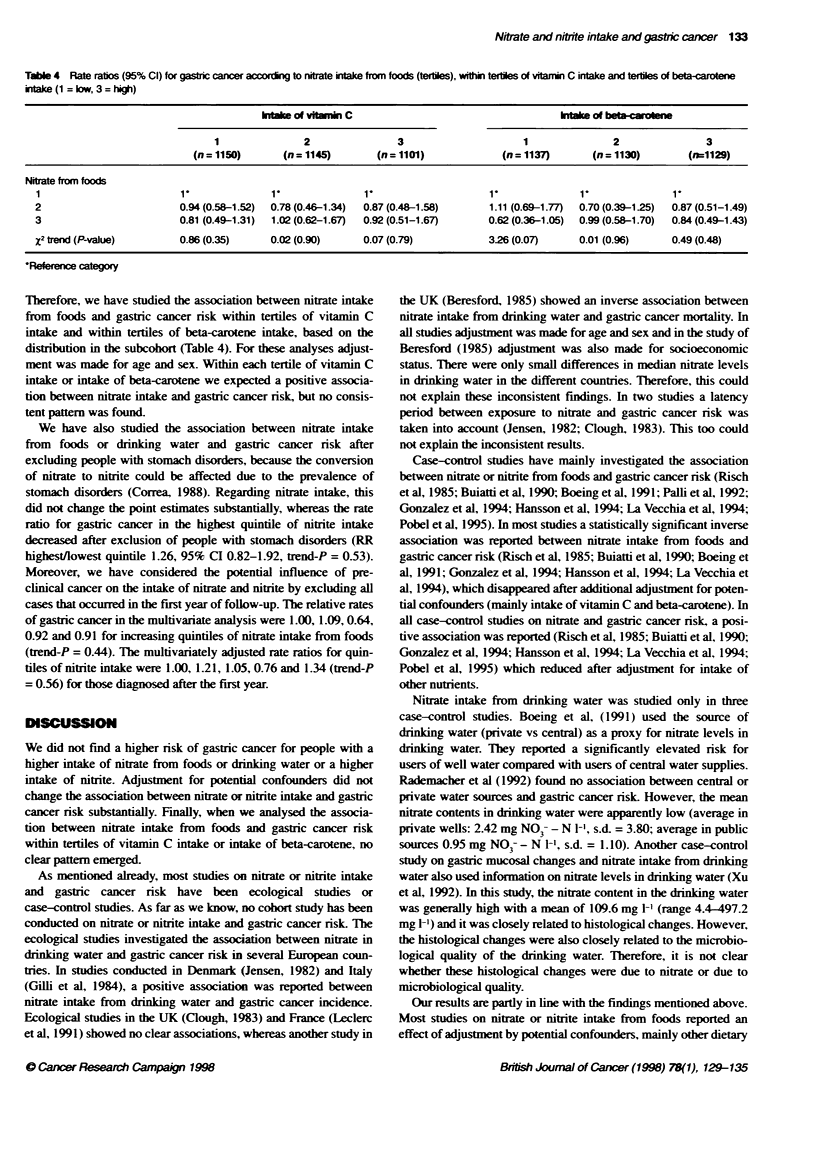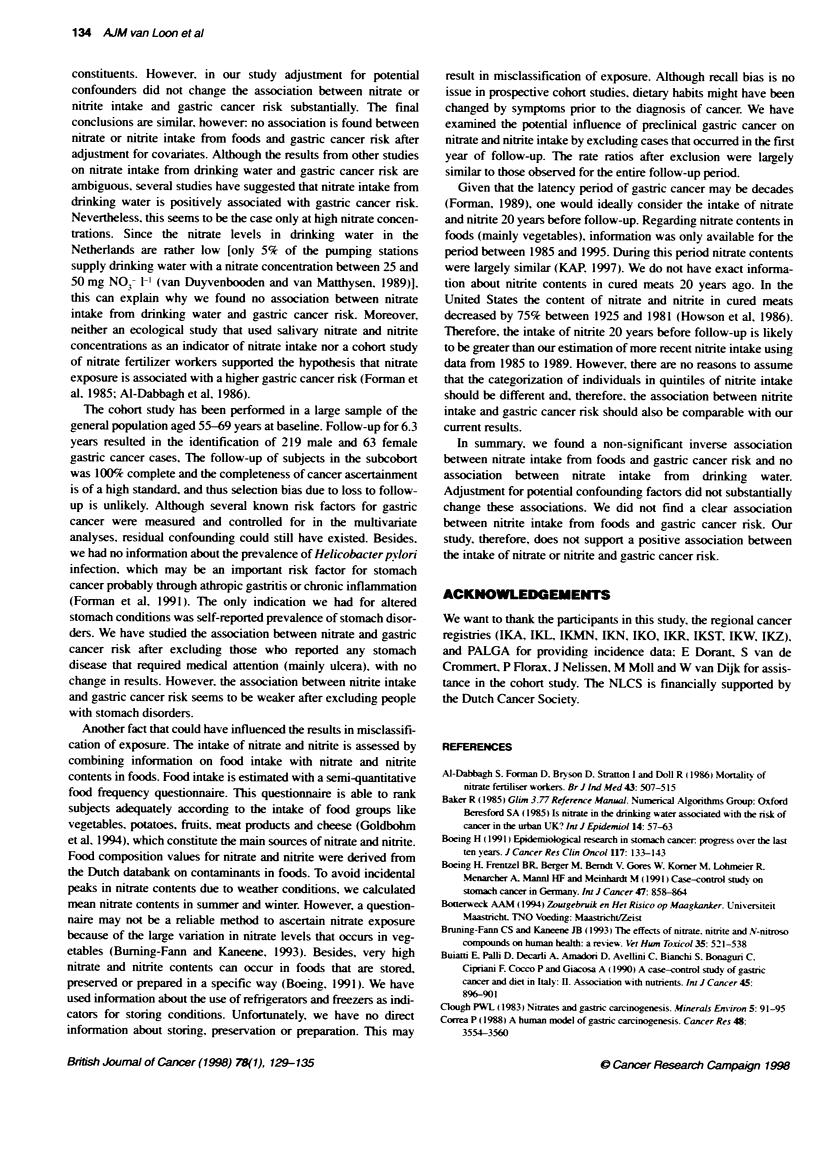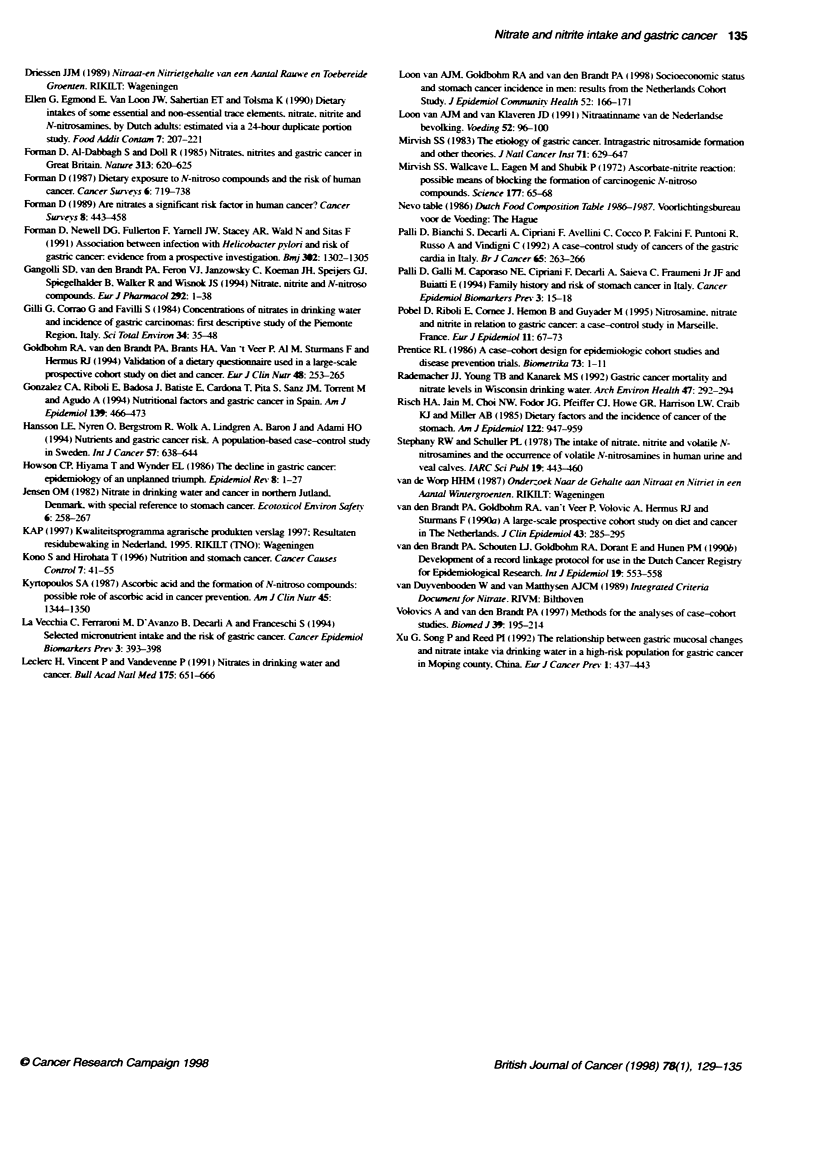# Intake of nitrate and nitrite and the risk of gastric cancer: a prospective cohort study.

**DOI:** 10.1038/bjc.1998.454

**Published:** 1998-07

**Authors:** A. J. van Loon, A. A. Botterweck, R. A. Goldbohm, H. A. Brants, J. D. van Klaveren, P. A. van den Brandt

**Affiliations:** University Masstricht, Department of Epidemiology, The Netherlands.

## Abstract

The association between the intake of nitrate or nitrite and gastric cancer risk was investigated in a prospective cohort study started in 1986 in the Netherlands, of 120,852 men and women aged 55-69 years. At baseline, data on dietary intake, smoking habits and other covariates were collected by means of a self-administered questionnaire. For data analysis, a case-cohort approach was used, in which the person-years at risk were estimated from a randomly selected subcohort (1688 men and 1812 women). After 6.3 years of follow-up, 282 microscopically confirmed incident cases of stomach cancer were detected: 219 men and 63 women. We did not find a higher risk of gastric cancer among people with a higher nitrate intake from food [rate ratio (RR) highest/lowest quintile = 0.80, 95% CI 0.47-1.37, trend-P = 0.18], a higher nitrate intake from drinking water (RR highest/lowest quintile = 0.88, 95% CI 0.59-1.32, trend-P = 0.39) or a higher intake of nitrite (RR highest/lowest quintile = 1.44, 95% CI 0.95-2.18, trend-P = 0.24). Rate ratios for gastric cancer were also computed for each tertile of nitrate intake from foods within tertiles of vitamin C intake and intake of beta-carotene, but no consistent pattern was found. Therefore, our study does not support a positive association between the intake of nitrate or nitrite and gastric cancer risk.


British Journal of Cancer (1998) 78(1), 129–135
© 1998 Cancer Research Campaign

### Intake of nitrate and nitrite and the risk of gastric cancer: a prospective cohort study

**AJM van Loon[1], AAM Botterweck[1], RA Goldbohm[2], HAM Brants[2], JD van Klaveren[3] and PA van den Brandt[1]**

[1]University Maastricht, Department of Epidemiology, PO Box 616, 6200 MD, Maastricht, The Netherlands; [2]TNO Nutrition and Food Research Institute, Department of Consumer Research and Epidemiology, PO Box 360, 3700 AJ, Zeist, The Netherlands; [3]State Institute for Quality Control of Agricultural Products, PO Box 230, 6700 AE, Wageningen, The Netherlands

**Summary** The association between the intake of nitrate or nitrite and gastric cancer risk was investigated in a prospective cohort study started in 1986 in the Netherlands, of 120 852 men and women aged 55–69 years. At baseline, data on dietary intake, smoking habits and other covariates were collected by means of a self-administered questionnaire. For data analysis, a case–cohort approach was used, in which the person–years at risk were estimated from a randomly selected subcohort (1688 men and 1812 women). After 6.3 years of follow-up, 282 microscopically confirmed incident cases of stomach cancer were detected: 219 men and 63 women. We did not find a higher risk of gastric cancer among people with a higher nitrate intake from food [rate ratio (RR) highest/lowest quintile = 0.80, 95% CI 0.47–1.37, trend-$P$ = 0.18], a higher nitrate intake from drinking water (RR highest/lowest quintile = 0.88, 95% CI 0.59–1.32, trend-$P$ = 0.39) or a higher intake of nitrite (RR highest/lowest quintile = 1.44, 95% CI 0.95–2.18, trend-$P$ = 0.24). Rate ratios for gastric cancer were also computed for each tertile of nitrate intake from foods within tertiles of vitamin C intake and intake of beta-carotene, but no consistent pattern was found. Therefore, our study does not support a positive association between the intake of nitrate or nitrite and gastric cancer risk.

**Keywords:** gastric cancer; nitrate; nitrite; dietary; drinking water; cohort study

Over the past 20 years there has been an accumulation of nitrate in vegetables due to the methods of cultivation. Moreover, nitrate content in drinking water has been increasing as a consequence of the extensive use of artificial fertilizers. This accumulation of nitrate has again raised the question whether high intake of nitrate leads to specific health risks, especially gastric cancer (Gangolli et al, 1994). In the Netherlands, the main sources of nitrate are leafy and other vegetables, potatoes and drinking water (van Loon and van Klaveren, 1991). In addition, both nitrite and nitrate are used as food additives in cheese and cured meats. It is not nitrate per se, but metabolites of nitrate, which are known carcinogens. Nitrate can be converted into nitrite, which can react with secondary amines or amides to produce carcinogenic *N*-nitroso compounds. In the Netherlands, estimations of the nitrate intake vary between 52 mg nitrate ion day$^{-1}$ (Ellen et al, 1990) and 131 mg nitrate ion day$^{-1}$ (Stephany and Schuller, 1978). The intake of nitrite is estimated as between 0.1 μg nitrite ion per day (Ellen et al, 1990) and 5.2 mg nitrite ion day$^{-1}$ (Stephany and Schuller, 1978). As about 5% of all ingested nitrate is converted to nitrite (Forman, 1987), there is usually a greater exposure to nitrite from the reduction of nitrate than from exogenous intake. Approximately 20% of the nitrite that enters the stomach arises directly from nitrite in the diet and 80% arises from the reduction of salivary nitrate (Mirvish, 1983). Nevertheless, dietary nitrite increases gastric nitrite levels when nitrosatable compounds are ingested, whereas this situation is only partly true for salivary nitrite (Mirvish, 1983). Therefore, it is relevant to study the intake of both nitrate and nitrite in relation to gastric cancer risk.

*Received 6 October 1997*
*Revised 3 December 1997*
*Accepted 7 January 1998*

*Correspondence to:* AAM Botterweck

Several factors can influence the conversion of nitrate to nitrite and *N*-nitroso compounds. Ascorbic acid can inhibit nitrosation by acting as a competitive substrate for nitrite (Mirvish et al, 1972). Both ascorbic acid and beta-carotene may act as scavengers for free radicals, thus preventing oxidative damage in gastric mucosa and mutations in DNA (Kyrtopoulos, 1987). Furthermore, the conversion of nitrate to nitrite may be inhibited by storage of foods in the refrigerator or freezer (Boeing, 1991). Other factors such as smoking habits, socioeconomic status, family history of stomach cancer and prevalence of stomach disorders may confound the association between nitrate or nitrite intake and gastric cancer risk (Boeing et al, 1991; Palli et al, 1994; Kono and Hirohata, 1996; van Loon et al, 1998). Numerous epidemiological studies which have been undertaken on the association between nitrate intake and gastric cancer risk show a lack of consistency (Jensen, 1982; Clough, 1983; Gilli et al, 1984; Beresford, 1985; Risch et al, 1985; Buiatti et al, 1990; Boeing et al, 1991; Leclerc et al, 1991; Palli et al, 1992; Rademacher et al, 1992; Xu et al, 1992; Gonzalez et al, 1994; Hansson et al, 1994; La Vecchia et al, 1994; Pobel et al, 1995). However, in most of these studies high nitrate consumption via ingested food showed no association with gastric cancer risk or even an inverse association. This finding might result from the fact that vegetables – the main source of nitrate – also contain vitamin C and beta-carotene, which appear to be protective factors for gastric cancer. Therefore, intake of nitrate from foods must be studied separately from intake of nitrate from drinking water, which does not contain protective substances.

So far, epidemiological studies on nitrate intake and gastric cancer risk have been ecological or case–control in design, in which problems arise with control for confounding, the long latency period and the accurate recall of information on food intakes. Some of these problems can be overcome in prospective cohort studies. The Netherlands Cohort Study (NLCS) is a prospective study on diet and cancer in which most relevant information was available to study the association between the intake of nitrate (from foods and drinking water) and the intake of nitrite and gastric cancer risk, with control for potential confounders.

### MATERIALS AND METHODS

### The Cohort Study

The NLCS on diet, other lifestyle characteristics and cancer risk started in September 1986. The cohort included 58 279 men and 62 573 women aged 55–69 years at the beginning of the study. Data were collected by means of a self-administered questionnaire. A detailed description of the cohort study design has been reported elsewhere (van den Brandt et al, 1990*a*). For data analysis, the case–cohort approach was used, in which gastric cancer cases were derived from the entire cohort (cohort cases), whereas the person–years at risk were estimated from a random sample of 3500 subjects (subcohort). After the baseline exposure measurement, the subcohort was randomly sampled (1688 men and 1812 women) and was followed up biennially for vital status information. Follow-up for incident cancer has been established by record linkage with all regional cancer registries in the Netherlands and with a national pathology register (PALGA). The method of record linkage has been described previously (van den Brandt et al, 1990*b*). The analysis is restricted to gastric cancer incidence in the period from September 1986 to December 1992. During these 6.3 years of cohort follow-up, 347 stomach cancer cases were detected. We excluded self-reported prevalent cancer cases other than skin cancer ($n = 33$), cases with in situ carcinoma ($n = 2$) and cases without microscopically confirmed diagnosis ($n = 2$). Therefore, 310 incident cohort cases (242 males and 68 females) were available for analysis. Self-reported prevalent cancer cases other than skin cancer were also excluded from the subcohort, with the result that 3346 subjects (1630 men and 1716 women) remained in this group. Finally, subjects with incomplete or inconsistent dietary data were excluded, leaving 282 cohort cases (219 men and 63 women) and 3123 subcohort members (1525 men and 1598 women) available for the analyses.

### Intake of nitrate and nitrite

The participants, usual consumption of food and beverages during the year preceding the start of the study was assessed at baseline with a 150-item semiquantitative food frequency questionnaire. This questionnaire had been validated against a 9-day diet record (Goldbohm et al, 1994) and it covered the main sources of nitrate (vegetables, drinking water) and nitrite (cured meat). Food composition values for nitrate were derived from the databank on contaminants in food from the State Institute for Quality Control of Agricultural Products (RIKILT, Wageningen). Estimations were based on the mean nitrate contents between 1985 and 1989, in which, for some vegetables (e.g. endive, lettuce), distinction was made between summer and winter. Furthermore, information on nitrate losses during preparation (washing, cutting and cooking) were considered. For several vegetables (endive, spinach, cabbage) and for potatoes, experimental data were available regarding nitrate losses during preparation (van de Worp, 1987; Driessen, 1989). For other vegetables nitrate losses were estimated to be 40%. Regarding nitrate intake from drinking water, we have combined information about nitrate contents in drinking water for each pumping station in the Netherlands in 1986 (databank on contaminants in food) with information about the distribution of drinking water (collected from all waterworks in the Netherlands). In this way, we could determine the nitrate content in drinking water for each home address by postal code. To calculate the nitrate intake from water, we also used information about the amount of water, coffee, tea and soup consumed (derived from the questionnaire). Food composition values for nitrite were obtained from TNO Nutrition and Food Research Institute (Zeist). Nitrite contents in vegetables and cheese were considered to be too low to include in the analyses. Therefore, nitrite intake was assessed solely on the intake of cured meat.

**Table 1** Distribution of nitrate and nitrite intake in gastric cancer cases and subcohort members

| | Subcohort members[a] | Gastric cancer cases[a] |
|---|---|---|
| Total | 3123 | 282 |
| Sex (% male) | 48.8 | 77.7 |
| Age (mean ± s.d.) | 61.4 ± 4.2 | 63.0 ± 4.1 |
| Level of education (% primary school only) | 30.1 | 37.4 |
| Stomach disorders (% ever) | 9.3 | 19.1 |
| Family history stomach cancer (% yes) | 6.7 | 11.3 |
| Refrigerator (mean number years ± s.d.) | 24.8 ± 8.9 | 25.5 ± 8.9 |
| Freezer (% never) | 36.0 | 34.9 |
| Smoking (% never) | 34.9 | 15.6 |
| Smoking (% current) | 31.3 | 44.7 |
| Coffee intake (% > 4 cups day$^{-1}$) | 10.2 | 16.0 |
| Beta-carotene (mean mg equivalent vitamin A day$^{-1}$ ± s.d.) | 0.42 ± 0.24 | 0.40 ± 0.22 |
| Vitamin C (mean mg day$^{-1}$ ± s.d.) | 103 ± 43 | 97 ± 44 |
| Mean mg day$^{-1}$ ± s.d. | | |
| Dietary nitrate | 105 ± 44 | 102 ± 43 |
| Nitrate from drinking water | 5.8 ± 6.5 | 6.1 ± 7.2 |
| Total nitrate | 111 ± 45 | 108 ± 44 |
| Nitrite | 0.13 ± 0.14 | 0.15 ± 0.16 |

[a]Only subjects with complete dietary data.

© Cancer Research Campaign 1998

**Table 2** Association between possible confounders and nitrate or nitrite intake in the subcohort members[1]

| | Nitrate (dietary): quintiles | | | | | Nitrate (drinking water): quintiles | | | | | Nitrite: quintiles | | | | |
|---|---|---|---|---|---|---|---|---|---|---|---|---|---|---|---|
| | I (low) | II | III | IV | V(high) | I (low) | II | III | IV | V(high) | I (low) | II | III | IV | V(high) |
| Sex (% male) | 47.4 | 47.5 | 49.8 | 49.5 | 49.9 | 47.0 | 48.1 | 49.9 | 52.3 | 46.8 | 39.3 | 40.4 | 47.5 | 54.3 | 62.8 |
| Age (mean) | 61.6 | 61.2 | 61.4 | 61.3 | 61.4 | 61.5 | 61.3 | 61.1 | 61.6 | 61.5 | 61.9 | 61.5 | 61.3 | 61.2 | 61.1 |
| Level of education (% primary school only) | 36.2 | 32.5 | 27.4 | 25.6 | 28.1 | 33.7 | 31.1 | 28.0 | 26.1 | 31.5 | 33.1 | 29.7 | 26.2 | 29.0 | 32.4 |
| Stomach disorders (% ever) | 12.3 | 8.5 | 10.1 | 7.0 | 8.7 | 8.3 | 9.2 | 9.5 | 10.4 | 9.1 | 8.8 | 7.3 | 8.8 | 11.3 | 10.4 |
| Family history stomach cancer (% yes) | 7.9 | 6.9 | 5.6 | 7.5 | 5.8 | 6.3 | 6.3 | 6.2 | 6.2 | 8.7 | 9.4 | 7.2 | 5.9 | 6.1 | 5.0 |
| Refrigerator (mean number of years) | 24.0 | 24.2 | 24.8 | 25.0 | 25.8 | 24.2 | 24.3 | 25.5 | 24.9 | 24.9 | 24.0 | 25.1 | 24.6 | 25.2 | 24.9 |
| Freezer (% never) | 36.6 | 35.2 | 34.8 | 38.0 | 35.4 | 29.5 | 33.7 | 38.3 | 38.1 | 40.6 | 40.7 | 35.8 | 35.0 | 35.2 | 33.2 |
| Smoking (% never) | 33.5 | 36.0 | 35.4 | 35.3 | 34.3 | 37.0 | 39.0 | 34.3 | 31.5 | 32.7 | 40.1 | 39.6 | 32.9 | 32.2 | 29.8 |
| Coffee intake (% > 4 cups day$^{-1}$) | 12.8 | 8.6 | 9.0 | 10.1 | 10.3 | 11.5 | 6.1 | 7.3 | 10.6 | 15.2 | 10.1 | 8.1 | 8.9 | 8.8 | 14.9 |
| Beta-carotene (mean mg eq vit A) | 0.23 | 0.32 | 0.38 | 0.47 | 0.70 | 0.42 | 0.42 | 0.41 | 0.43 | 0.43 | 0.43 | 0.41 | 0.40 | 0.42 | 0.44 |
| Vitamin C (mean mg day$^{-1}$) | 75.9 | 90.0 | 100.6 | 114.4 | 136.2 | 99.0 | 101.2 | 103.0 | 105.1 | 108.9 | 102.5 | 104.1 | 100.4 | 105.1 | 105.1 |

[1]Only subjects with complete dietary data

### Other relevant aspects

Other factors relevant to the association between nitrate or nitrite intake and gastric cancer risk that were measured were smoking status (never/ex/current), level of education (primary school, lower vocational school, junior high school, senior high school, higher vocational school or university), family history of stomach cancer (yes or no), prevalence of stomach disorders (any stomach disease in the past that required medical attention), the use of refrigerator (number of years) or freezer (yes or no) and the intake of coffee (categorical), vitamin C (mg day$^{-1}$) and beta-carotene (mg day$^{-1}$). Mean individual nutrient intakes per day were computed using the Dutch food table of 1986 (Nevo, 1986).

Although high salt intake is linked with increased risk for gastric cancer in many aetiological studies (Boeing, 1991), preliminary results from the NLCS showed no association between salt intake and gastric cancer risk (Botterweck, 1994). Salt intake was therefore, omitted from the analyses.

### Data analysis

The intake of nitrate from different sources, the intake of nitrite and the distribution of potential confounders possibly associated with nitrate or nitrite intake and gastric cancer risk were compared between the cohort cases and subcohort group. Intake of nitrate and nitrite was categorized into quintiles according to the distribution in the subcohort. The associations between nitrate intake or nitrite intake and covariates were studied in the subcohort by comparing the distribution of several covariates among the quintiles of nitrate intake from foods, nitrate intake from drinking water and intake of nitrite. To study the association between intake of nitrate and nitrite and gastric cancer risk and the role of possible confounders, data were analysed according to the case–cohort approach (Prentice, 1986; Volovics and van den Brandt, 1997) using the GLIM statistical package (Baker, 1985). First, age- and sex-adjusted rate ratios (RRs) and 95% confidence intervals (CIs) for gastric cancer were determined for nitrate intake from foods, nitrate intake from drinking water, total intake of nitrate and intake of nitrite. In the multivariate analyses, rate ratios for gastric cancer were computed for the different exposures after adjustment for age, sex, smoking status, highest level of education, intake of vitamin C and beta-carotene, family history of stomach cancer, prevalence of stomach disorders and use of refrigerator or freezer. To distinguish possible positive associations between nitrate intake from foods and gastric cancer risk from possible effects of the intake of vitamin C or beta-carotene on gastric cancer risk, we have computed rate ratios for gastric cancer for each tertile of nitrate intake from foods within tertiles of vitamin C intake and intake of beta-carotene. Moreover, the association between nitrate intake from foods or drinking water and gastric cancer risk was studied after exclusion of people with self-reported stomach disorders, because the conversion of nitrate to nitrite could be influenced by the prevalence of stomach disorders (Correa, 1988). Finally, analyses were also conducted after excluding cases that occurred in the first year of follow-up to consider the potential influence of preclinical gastric cancer on the intake of nitrate or nitrite.

### RESULTS

The mean intake of nitrate and nitrite and the distribution of relevant covariates in the group of cohort cases and subcohort members is presented in Table 1. Among the cohort cases, there were proportionally more men compared with the subcohort members and cohort cases were on average older than members of the subcohort (mean age for cohort cases 63.0 years and for subcohort members 61.4 years). A higher percentage of the cohort cases had primary school as the highest level of education compared with subcohort members. Stomach disorders were more prevalent among cohort cases compared with members of the subcohort. The same was found with regard to family history of stomach cancer. The mean number of years of refrigerator use was only slightly higher among cohort cases, although proportionally fewer cohort cases used a freezer, compared with the use of refrigerator and freezer by the subcohort members. There were fewer non-smokers among cohort cases and more current smokers compared with smoking habits of the subcohort members and proportionally more cohort cases consumed large amounts of coffee (>4 cups per day) than subcohort members. The mean intake of beta-carotene did not differ substantially between cohort cases and subcohort members and the intake of vitamin C was somewhat lower among cohort cases. The intake of nitrate from foods, nitrate from drinking water and nitrite did not substantially differ between cohort cases and subcohort members; cohort cases had a somewhat higher intake of nitrate from drinking water and a slightly higher intake of nitrite, while the intake of nitrate from foods and the total nitrate intake was somewhat lower among cohort cases compared with members of the subcohort.

© Cancer Research Campaign 1998

**Table 3** Rate ratios for gastric cancer according to nitrate and nitrite intake (quintiles)

| | No. of cases in cohort | Person-years in subcohort | RR (95% CI)[1] | RR (95% CI)[2] |
|---|---|---|---|---|
| Dietary nitrate (mean, mg day$^{-1}$) | | | | |
| I (55.8) | 69 | 3784 | 1* | 1* |
| II (79.4) | 61 | 3813 | 0.93 (0.64–1.33) | 1.02 (0.69–1.51) |
| III (98.7) | 45 | 3814 | 0.65 (0.44–0.96) | 0.71 (0.46–1.09) |
| IV (120.7) | 49 | 3813 | 0.71 (0.48–1.04) | 0.80 (0.51–1.25) |
| V (172.2) | 58 | 3796 | 0.83 (0.58–1.20) | 0.80 (0.47–1.37) |
| $\chi^2$trend test (*P*-value) | | | 2.55 (0.11) | 1.82 (0.18) |
| Nitrate from drinking water (mean, mg day$^{-1}$) | | | | |
| I (0.02) | 61 | 3836 | 1* | 1* |
| II (1.65) | 54 | 3790 | 0.91 (0.62–1.34) | 0.93 (0.62–1.39) |
| III (3.85) | 53 | 3829 | 0.87 (0.59–1.28) | 0.87 (0.58–1.31) |
| IV (6.91) | 57 | 3812 | 0.86 (0.59–1.27) | 0.83 (0.55–1.24) |
| V (16.5) | 57 | 3750 | 0.94 (0.64–1.38) | 0.88 (0.59–1.32) |
| $\chi^2$trend test (*P*-value) | | | 0.20 (0.66) | 0.73 (0.39) |
| Total nitrate (mean, mg day$^{-1}$) | | | | |
| I (59.8) | 63 | 3771 | 1* | 1* |
| II (84.7) | 67 | 3844 | 1.11 (0.77–1.60) | 1.25 (0.84–1.86) |
| III (104.4) | 42 | 3805 | 0.65 (0.43–0.98) | 0.74 (0.47–1.15) |
| IV (127.3) | 54 | 3820 | 0.83 (0.57–1.22) | 0.92 (0.59–1.44) |
| V (179.8) | 56 | 3779 | 0.88 (0.60–1.28) | 0.90 (0.53–1.55) |
| $\chi^2$trend test (*P*-value) | | | 1.85 (0.17) | 1.08 (0.30) |
| Nitrite (mean, mg day$^{-1}$) | | | | |
| I (0.01) | 47 | 3873 | 1* | 1* |
| II (0.04) | 51 | 3706 | 1.15 (0.76–1.74) | 1.20 (0.78–1.86) |
| III (0.09) | 58 | 3829 | 1.21 (0.81–1.83) | 1.18 (0.77–1.82) |
| IV (0.16) | 46 | 3844 | 0.87 (0.57–1.33) | 0.88 (0.56–1.37) |
| V (0.35) | 80 | 3760 | 1.49 (1.01–2.20) | 1.44 (0.95–2.18) |
| $\chi^2$ trend test (*P*-value) | | | 2.20 (0.14) | 1.38 (0.24) |

*Reference category. [1]Adjustment for age and sex. [2]Multivariate analyses with adjustment for age, sex, smoking (never/ex/current), highest level of education, coffee consumption, intake of vitamin C and beta-carotene, family history of stomach cancer (yes or no), prevalence of stomach disorders (ever or never), use of refrigerator (number of years) and use of freezer (ever or never).

The association between covariates and nitrate intake from foods, nitrate intake from drinking water or nitrite intake was studied in the subcohort (Table 2). Proportionally, more men were found in the higher quintiles of nitrate intake and proportionally more people with only primary school education were found in the lowest quintile of nitrate intake from foods. Also, a correlation was found between the prevalence of stomach disorders and nitrate intake from foods. Moreover, nitrate intake from foods was positively correlated with the intake of beta-carotene and vitamin C. Nitrate intake from drinking water was positively correlated with use of freezer (% never), coffee consumption (%> 4 cups per day) and intake of vitamin C. Regarding nitrite intake, proportionally more men were found in the higher quintiles and the percentage of never smokers was higher within the lower quintiles of nitrite intake. Furthermore, nitrite intake was inversely correlated with family history of stomach cancer (% ever) and use of freezer (% never).

The results of the age- and sex-adjusted analyses are presented in Table 3. There was a non-significant inverse association between nitrate intake from foods and gastric cancer risk (RR highest/lowest quintile = 0.83, 95% CI 0.58–1.20, trend-*P* = 0.11) and also between the total nitrate intake and gastric cancer risk (RR highest/lowest quintile = 0.88, 95% CI 0.60–1.28, trend-*P* = 0.17). No association was found between nitrate intake from drinking water and gastric cancer risk (RR highest/lowest quintile = 0.94, 95% CI 0.64–1.38, trend-*P* = 0.66). The association between nitrite intake and gastric cancer risk was not clear. The significantly higher risk for gastric cancer was found in the highest quintile of nitrite intake (RR = 1.49, 95% CI 1.01–2.20), but the gastric cancer risk in the second highest quintile was below one (RR = 0.87, 95% CI 0.57–1.33). In the multivariate analyses, adjustment was made for age, sex, smoking status, highest level of education, coffee consumption, intake of vitamin C and beta-carotene, family history of stomach cancer, prevalence of stomach disorders, use of refrigerator and use of freezer (Table 3). After adjustment, there was still a non-significant inverse association between nitrate intake from foods and gastric cancer risk (RR highest/lowest quintile = 0.80, 95% CI 0.47–1.37, trend-*P* = 0.18). Nor did the association between total nitrate intake and gastric cancer risk change after additional adjustment (RR highest/lowest quintile = 0.90, 95% CI 0.53–1.55, trend-*P* = 0.30). Regarding nitrate intake from drinking water and gastric cancer risk, the rate ratio in the highest quintile changed marginally to 0.88 (95% CI = 0.59–1.32, trend-*P* = 0.39). The association between nitrite intake and gastric cancer risk was still ambiguous.

The non-significant inverse association between nitrate intake from foods and gastric cancer risk was thought to be due to a protective effect of vegetables like vitamin C and beta-carotene.

© Cancer Research Campaign 1998

**Table 4** Rate ratios (95% CI) for gastric cancer according to nitrate intake from foods (tertiles), within tertiles of vitamin C intake and tertiles of beta-carotene intake (1 = low, 3 = high)

| | Intake of vitamin C | | | Intake of beta-carotene | | |
|---|---|---|---|---|---|---|
| | 1 ($n$ = 1150) | 2 ($n$ = 1145) | 3 ($n$ = 1101) | 1 ($n$ = 1137) | 2 ($n$ = 1130) | 3 ($n$=1129) |
| Nitrate from foods | | | | | | |
| 1 | 1* | 1* | 1* | 1* | 1* | 1* |
| 2 | 0.94 (0.58–1.52) | 0.78 (0.46–1.34) | 0.87 (0.48–1.58) | 1.11 (0.69–1.77) | 0.70 (0.39–1.25) | 0.87 (0.51–1.49) |
| 3 | 0.81 (0.49–1.31) | 1.02 (0.62–1.67) | 0.92 (0.51–1.67) | 0.62 (0.36–1.05) | 0.99 (0.58–1.70) | 0.84 (0.49–1.43) |
| $\chi^2$ trend ($P$-value) | 0.86 (0.35) | 0.02 (0.90) | 0.07 (0.79) | 3.26 (0.07) | 0.01 (0.96) | 0.49 (0.48) |

*Reference category

Therefore, we have studied the association between nitrate intake from foods and gastric cancer risk within tertiles of vitamin C intake and within tertiles of beta-carotene intake, based on the distribution in the subcohort (Table 4). For these analyses adjustment was made for age and sex. Within each tertile of vitamin C intake or intake of beta-carotene we expected a positive association between nitrate intake and gastric cancer risk, but no consistent pattern was found.

We have also studied the association between nitrate intake from foods or drinking water and gastric cancer risk after excluding people with stomach disorders, because the conversion of nitrate to nitrite could be affected due to the prevalence of stomach disorders (Correa, 1988). Regarding nitrate intake, this did not change the point estimates substantially, whereas the rate ratio for gastric cancer in the highest quintile of nitrite intake decreased after exclusion of people with stomach disorders (RR highest/lowest quintile 1.26, 95% CI 0.82–1.92, trend-$P$ = 0.53). Moreover, we have considered the potential influence of preclinical cancer on the intake of nitrate and nitrite by excluding all cases that occurred in the first year of follow-up. The relative rates of gastric cancer in the multivariate analysis were 1.00, 1.09, 0.64, 0.92 and 0.91 for increasing quintiles of nitrate intake from foods (trend-$P$ = 0.44). The multivariately adjusted rate ratios for quintiles of nitrite intake were 1.00, 1.21, 1.05, 0.76 and 1.34 (trend-$P$ = 0.56) for those diagnosed after the first year.

### DISCUSSION

We did not find a higher risk of gastric cancer for people with a higher intake of nitrate from foods or drinking water or a higher intake of nitrite. Adjustment for potential confounders did not change the association between nitrate or nitrite intake and gastric cancer risk substantially. Finally, when we analysed the association between nitrate intake from foods and gastric cancer risk within tertiles of vitamin C intake or intake of beta-carotene, no clear pattern emerged.

As mentioned already, most studies on nitrate or nitrite intake and gastric cancer risk have been ecological studies or case–control studies. As far as we know, no cohort study has been conducted on nitrate or nitrite intake and gastric cancer risk. The ecological studies investigated the association between nitrate in drinking water and gastric cancer risk in several European countries. In studies conducted in Denmark (Jensen, 1982) and Italy (Gilli et al, 1984), a positive association was reported between nitrate intake from drinking water and gastric cancer incidence. Ecological studies in the UK (Clough, 1983) and France (Leclerc et al, 1991) showed no clear associations, whereas another study in the UK (Beresford, 1985) showed an inverse association between nitrate intake from drinking water and gastric cancer mortality. In all studies adjustment was made for age and sex and in the study of Beresford (1985) adjustment was also made for socioeconomic status. There were only small differences in median nitrate levels in drinking water in the different countries. Therefore, this could not explain these inconsistent findings. In two studies a latency period between exposure to nitrate and gastric cancer risk was taken into account (Jensen, 1982; Clough, 1983). This too could not explain the inconsistent results.

Case–control studies have mainly investigated the association between nitrate or nitrite from foods and gastric cancer risk (Risch et al, 1985; Buiatti et al, 1990; Boeing et al, 1991; Palli et al, 1992; Gonzalez et al, 1994; Hansson et al, 1994; La Vecchia et al, 1994; Pobel et al, 1995). In most studies a statistically significant inverse association was reported between nitrate intake from foods and gastric cancer risk (Risch et al, 1985; Buiatti et al, 1990; Boeing et al, 1991; Gonzalez et al, 1994; Hansson et al, 1994; La Vecchia et al, 1994), which disappeared after additional adjustment for potential confounders (mainly intake of vitamin C and beta-carotene). In all case–control studies on nitrate and gastric cancer risk, a positive association was reported (Risch et al, 1985; Buiatti et al, 1990; Gonzalez et al, 1994; Hansson et al, 1994; La Vecchia et al, 1994; Pobel et al, 1995) which reduced after adjustment for intake of other nutrients.

Nitrate intake from drinking water was studied only in three case–control studies. Boeing et al, (1991) used the source of drinking water (private vs central) as a proxy for nitrate levels in drinking water. They reported a significantly elevated risk for users of well water compared with users of central water supplies. Rademacher et al (1992) found no association between central or private water sources and gastric cancer risk. However, the mean nitrate contents in drinking water were apparently low (average in private wells: 2.42 mg $NO_3^-$ – N $l^{-1}$, s.d. = 3.80; average in public sources 0.95 mg $NO_3^-$ – N $l^{-1}$, s.d. = 1.10). Another case–control study on gastric mucosal changes and nitrate intake from drinking water also used information on nitrate levels in drinking water (Xu et al, 1992). In this study, the nitrate content in the drinking water was generally high with a mean of 109.6 mg $l^{-1}$ (range 4.4–497.2 mg $l^{-1}$) and it was closely related to histological changes. However, the histological changes were also closely related to the microbiological quality of the drinking water. Therefore, it is not clear whether these histological changes were due to nitrate or due to microbiological quality.

Our results are partly in line with the findings mentioned above. Most studies on nitrate or nitrite intake from foods reported an effect of adjustment by potential confounders, mainly other dietary constituents. However, in our study adjustment for potential confounders did not change the association between nitrate or nitrite intake and gastric cancer risk substantially. The final conclusions are similar, however: no association is found between nitrate or nitrite intake from foods and gastric cancer risk after adjustment for covariates. Although the results from other studies on nitrate intake from drinking water and gastric cancer risk are ambiguous, several studies have suggested that nitrate intake from drinking water is positively associated with gastric cancer risk. Nevertheless, this seems to be the case only at high nitrate concentrations. Since the nitrate levels in drinking water in the Netherlands are rather low [only 5% of the pumping stations supply drinking water with a nitrate concentration between 25 and 50 mg $NO_3^-$ $l^{-1}$ (van Duyvenbooden and van Matthysen, 1989)], this can explain why we found no association between nitrate intake from drinking water and gastric cancer risk. Moreover, neither an ecological study that used salivary nitrate and nitrite concentrations as an indicator of nitrate intake nor a cohort study of nitrate fertilizer workers supported the hypothesis that nitrate exposure is associated with a higher gastric cancer risk (Forman et al, 1985; Al-Dabbagh et al, 1986).

The cohort study has been performed in a large sample of the general population aged 55–69 years at baseline. Follow-up for 6.3 years resulted in the identification of 219 male and 63 female gastric cancer cases. The follow-up of subjects in the subcobort was 100% complete and the completeness of cancer ascertainment is of a high standard, and thus selection bias due to loss to follow-up is unlikely. Although several known risk factors for gastric cancer were measured and controlled for in the multivariate analyses, residual confounding could still have existed. Besides, we had no information about the prevalence of *Helicobacter pylori* infection, which may be an important risk factor for stomach cancer probably through athropic gastritis or chronic inflammation (Forman et al, 1991). The only indication we had for altered stomach conditions was self-reported prevalence of stomach disorders. We have studied the association between nitrate and gastric cancer risk after excluding those who reported any stomach disease that required medical attention (mainly ulcera), with no change in results. However, the association between nitrite intake and gastric cancer risk seems to be weaker after excluding people with stomach disorders.

Another fact that could have influenced the results in misclassification of exposure. The intake of nitrate and nitrite is assessed by combining information on food intake with nitrate and nitrite contents in foods. Food intake is estimated with a semi-quantitative food frequency questionnaire. This questionnaire is able to rank subjects adequately according to the intake of food groups like vegetables, potatoes, fruits, meat products and cheese (Goldbohm et al, 1994), which constitute the main sources of nitrate and nitrite. Food composition values for nitrate and nitrite were derived from the Dutch databank on contaminants in foods. To avoid incidental peaks in nitrate contents due to weather conditions, we calculated mean nitrate contents in summer and winter. However, a questionnaire may not be a reliable method to ascertain nitrate exposure because of the large variation in nitrate levels that occurs in vegetables (Burning-Fann and Kaneene, 1993). Besides, very high nitrate and nitrite contents can occur in foods that are stored, preserved or prepared in a specific way (Boeing, 1991). We have used information about the use of refrigerators and freezers as indicators for storing conditions. Unfortunately, we have no direct information about storing, preservation or preparation. This may result in misclassification of exposure. Although recall bias is no issue in prospective cohort studies, dietary habits might have been changed by symptoms prior to the diagnosis of cancer. We have examined the potential influence of preclinical gastric cancer on nitrate and nitrite intake by excluding cases that occurred in the first year of follow-up. The rate ratios after exclusion were largely similar to those observed for the entire follow-up period.

Given that the latency period of gastric cancer may be decades (Forman, 1989), one would ideally consider the intake of nitrate and nitrite 20 years before follow-up. Regarding nitrate contents in foods (mainly vegetables), information was only available for the period between 1985 and 1995. During this period nitrate contents were largely similar (KAP, 1997). We do not have exact information about nitrite contents in cured meats 20 years ago. In the United States the content of nitrate and nitrite in cured meats decreased by 75% between 1925 and 1981 (Howson et al, 1986). Therefore, the intake of nitrite 20 years before follow-up is likely to be greater than our estimation of more recent nitrite intake using data from 1985 to 1989. However, there are no reasons to assume that the categorization of individuals in quintiles of nitrite intake should be different and, therefore, the association between nitrite intake and gastric cancer risk should also be comparable with our current results.

In summary, we found a non-significant inverse association between nitrate intake from foods and gastric cancer risk and no association between nitrate intake from drinking water. Adjustment for potential confounding factors did not substantially change these associations. We did not find a clear association between nitrite intake from foods and gastric cancer risk. Our study, therefore, does not support a positive association between the intake of nitrate or nitrite and gastric cancer risk.

### ACKNOWLEDGEMENTS

We want to thank the participants in this study, the regional cancer registries (IKA, IKL, IKMN, IKN, IKO, IKR, IKST, IKW, IKZ), and PALGA for providing incidence data; E Dorant, S van de Crommert, P Florax, J Nelissen, M Moll and W van Dijk for assistance in the cohort study. The NLCS is financially supported by the Dutch Cancer Society.

### REFERENCES

Al-Dabbagh S, Forman D, Bryson D, Stratton I and Doll R (1986) Mortality of nitrate fertiliser workers. *Br J Ind Med* **43**: 507–515

Baker R (1985) *Glim 3.77 Reference Manual*. Numerical Algorithms Group: Oxford

Beresford SA (1985) Is nitrate in the drinking water associated with the risk of cancer in the urban UK? *Int J Epidemiol* **14**: 57–63

Boeing H (1991) Epidemiological research in stomach cancer: progress over the last ten years. *J Cancer Res Clin Oncol* **117**: 133–143

Boeing H, Frentzel BR, Berger M, Berndt V, Gores W, Korner M, Lohmeier R, Menarcher A, Mannl HF and Meinhardt M (1991) Case–control study on stomach cancer in Germany. *Int J Cancer* **47**: 858–864

Botterweck AAM (1994) *Zoutgebruik en Het Risico op Maagkanker*. Universiteit Maastricht. TNO Voeding: Maastricht/Zeist

Bruning-Fann CS and Kaneene JB (1993) The effects of nitrate, nitrite and *N*-nitroso compounds on human health: a review. *Vet Hum Toxicol* **35**: 521–538

Buiatti E, Palli D, Decarli A, Amadori D, Avellini C, Bianchi S, Bonaguri C, Cipriani F, Cocco P and Giacosa A (1990) A case–control study of gastric cancer and diet in Italy: II. Association with nutrients. *Int J Cancer* **45**: 896–901

Clough PWL (1983) Nitrates and gastric carcinogenesis. *Minerals Environ* **5**: 91–95

Correa P (1988) A human model of gastric carcinogenesis. *Cancer Res* **48**: 3554–3560

© Cancer Research Campaign 1998

Driessen JJM (1989) *Nitraat-en Nitrietgehalte van een Aantal Rauwe en Toebereide Groenten*. RIKILT: Wageningen

Ellen G, Egmond E, Van Loon JW, Sahertian ET and Tolsma K (1990) Dietary intakes of some essential and non-essential trace elements, nitrate, nitrite and *N*-nitrosamines, by Dutch adults: estimated via a 24-hour duplicate portion study. *Food Addit Contam* **7**: 207–221

Forman D, Al-Dabbagh S and Doll R (1985) Nitrates, nitrites and gastric cancer in Great Britain. *Nature* **313**: 620–625

Forman D (1987) Dietary exposure to *N*-nitroso compounds and the risk of human cancer. *Cancer Surveys* **6**: 719–738

Forman D (1989) Are nitrates a significant risk factor in human cancer? *Cancer Surveys* **8**: 443–458

Forman D, Newell DG, Fullerton F, Yarnell JW, Stacey AR, Wald N and Sitas F (1991) Association between infection with *Helicobacter pylori* and risk of gastric cancer: evidence from a prospective investigation. *Bmj* **302**: 1302–1305

Gangolli SD, van den Brandt PA, Feron VJ, Janzowsky C, Koeman JH, Speijers GJ, Spiegelhalder B, Walker R and Wisnok JS (1994) Nitrate, nitrite and *N*-nitroso compounds. *Eur J Pharmacol* **292**: 1–38

Gilli G, Corrao G and Favilli S (1984) Concentrations of nitrates in drinking water and incidence of gastric carcinomas: first descriptive study of the Piemonte Region, Italy. *Sci Total Environ* **34**: 35–48

Goldbohm RA, van den Brandt PA, Brants HA, Van 't Veer P, Al M, Sturmans F and Hermus RJ (1994) Validation of a dietary questionnaire used in a large-scale prospective cohort study on diet and cancer. *Eur J Clin Nutr* **48**: 253–265

Gonzalez CA, Riboli E, Badosa J, Batiste E, Cardona T, Pita S, Sanz JM, Torrent M and Agudo A (1994) Nutritional factors and gastric cancer in Spain. *Am J Epidemiol* **139**: 466–473

Hansson LE, Nyren O, Bergstrom R, Wolk A, Lindgren A, Baron J and Adami HO (1994) Nutrients and gastric cancer risk. A population-based case–control study in Sweden. *Int J Cancer* **57**: 638–644

Howson CP, Hiyama T and Wynder EL (1986) The decline in gastric cancer: epidemiology of an unplanned triumph. *Epidemiol Rev* **8**: 1–27

Jensen OM (1982) Nitrate in drinking water and cancer in northern Jutland, Denmark, with special reference to stomach cancer. *Ecotoxicol Environ Safety* **6**: 258–267

KAP (1997) Kwaliteitsprogramma agrarische produkten verslag 1997: Resultaten residubewaking in Nederland, 1995. RIKILT (TNO): Wageningen

Kono S and Hirohata T (1996) Nutrition and stomach cancer. *Cancer Causes Control* **7**: 41–55

Kyrtopoulos SA (1987) Ascorbic acid and the formation of *N*-nitroso compounds: possible role of ascorbic acid in cancer prevention. *Am J Clin Nutr* **45**: 1344–1350

La Vecchia C, Ferraroni M, D'Avanzo B, Decarli A and Franceschi S (1994) Selected micronutrient intake and the risk of gastric cancer. *Cancer Epidemiol Biomarkers Prev* **3**: 393–398

Leclerc H, Vincent P and Vandevenne P (1991) Nitrates in drinking water and cancer. *Bull Acad Natl Med* **175**: 651–666

Loon van AJM, Goldbohm RA and van den Brandt PA (1998) Socioeconomic status and stomach cancer incidence in men: results from the Netherlands Cohort Study. *J Epidemiol Community Health* 52: 166–171

Loon van AJM and van Klaveren JD (1991) Nitraatinname van de Nederlandse bevolking. *Voeding* **52**: 96–100

Mirvish SS (1983) The etiology of gastric cancer. Intragastric nitrosamide formation and other theories. *J Natl Cancer Inst* **71**: 629–647

Mirvish SS, Wallcave L, Eagen M and Shubik P (1972) Ascorbate-nitrite reaction: possible means of blocking the formation of carcinogenic *N*-nitroso compounds. *Science* **177**: 65–68

Nevo table (1986) *Dutch Food Composition Table 1986–1987*. Voorlichtingsbureau voor de Voeding: The Hague

Palli D, Bianchi S, Decarli A, Cipriani F, Avellini C, Cocco P, Falcini F, Puntoni R, Russo A and Vindigni C (1992) A case–control study of cancers of the gastric cardia in Italy. *Br J Cancer* **65**: 263–266

Palli D, Galli M, Caporaso NE, Cipriani F, Decarli A, Saieva C, Fraumeni Jr JF and Buiatti E (1994) Family history and risk of stomach cancer in Italy. *Cancer Epidemiol Biomarkers Prev* **3**: 15–18

Pobel D, Riboli E, Cornee J, Hemon B and Guyader M (1995) Nitrosamine, nitrate and nitrite in relation to gastric cancer: a case–control study in Marseille, France. *Eur J Epidemiol* **11**: 67–73

Prentice RL (1986) A case–cohort design for epidemiologic cohort studies and disease prevention trials. *Biometrika* **73**: 1–11

Rademacher JJ, Young TB and Kanarek MS (1992) Gastric cancer mortality and nitrate levels in Wisconsin drinking water. *Arch Environ Health* **47**: 292–294

Risch HA, Jain M, Choi NW, Fodor JG, Pfeiffer CJ, Howe GR, Harrison LW, Craib KJ and Miller AB (1985) Dietary factors and the incidence of cancer of the stomach. *Am J Epidemiol* **122**: 947–959

Stephany RW and Schuller PL (1978) The intake of nitrate, nitrite and volatile *N*-nitrosamines and the occurrence of volatile *N*-nitrosamines in human urine and veal calves. *IARC Sci Publ* **19**: 443–460

van de Worp HHM (1987) *Onderzoek Naar de Gehalte aan Nitraat en Nitriet in een Aantal Wintergroenten*. RIKILT: Wageningen van den Brandt PA, Goldbohm RA, van't Veer P, Volovic A, Hermus RJ and Sturmans F (1990*a*) A large-scale prospective cohort study on diet and cancer in The Netherlands. *J Clin Epidemiol* **43**: 285–295

van den Brandt PA, Schouten LJ, Goldbohm RA, Dorant E and Hunen PM (1990*b*) Development of a record linkage protocol for use in the Dutch Cancer Registry for Epidemiological Research. *Int J Epidemiol* **19**: 553–558

van Duyvenbooden W and van Matthysen AJCM (1989) *Integrated Criteria Document for Nitrate*. RIVM: Bilthoven

Volovics A and van den Brandt PA (1997) Methods for the analyses of case–cohort studies. *Biomed J* **39**: 195–214

Xu G, Song P and Reed PI (1992) The relationship between gastric mucosal changes and nitrate intake via drinking water in a high-risk population for gastric cancer in Moping county, China. *Eur J Cancer Prev* **1**: 437–443

© Cancer Research Campaign 1998